# Advancements in Soft-Tissue Prosthetics Part B: The Chemistry of Imitating Life

**DOI:** 10.3389/fbioe.2020.00147

**Published:** 2020-04-23

**Authors:** Rena L. J. Cruz, Maureen T. Ross, Sean K. Powell, Maria A. Woodruff

**Affiliations:** Institute of Health and Biomedical Innovation, Queensland University of Technology, Brisbane, QLD, Australia

**Keywords:** prosthetic, prosthesis, polymer, silicone, additive manufacturing, maxillofacial

## Abstract

Each year, congenital defects, trauma or cancer often results in considerable physical disfigurement for many people worldwide. This adversely impacts their psychological, social and economic outlook, leading to poor life experiences and negative health outcomes. In many cases of soft tissue disfigurement, highly personalized prostheses are available to restore both aesthetics and function. As discussed in part A of this review, key to the success of any soft tissue prosthetic is the fundamental properties of the materials. This determines the maximum attainable level of aesthetics, attachment mechanisms, fabrication complexity, cost, and robustness. Since the early-mid 20th century, polymers have completely replaced natural materials in prosthetics, with advances in both material properties and fabrication techniques leading to significantly improved capabilities. In part A, we discussed the history of polymers in prosthetics, their ideal properties, and the application of polymers in prostheses for the ear, nose, eye, breast and finger. We also reviewed the latest developments in advanced manufacturing and 3D printing, including different fabrication technologies and new and upcoming materials. In this review, Part B, we detail the chemistry of the most commonly used synthetic polymers in soft tissue prosthetics; silicone, acrylic resin, vinyl polymer, and polyurethane elastomer. For each polymer, we briefly discuss their history before detailing their chemistry and fabrication processes. We also discuss degradation of the polymer in the context of their application in prosthetics, including time and weathering, the impact of skin secretions, microbial growth and cleaning and disinfecting. Although advanced manufacturing promises new fabrication capabilities using exotic synthetic polymers with programmable material properties, silicones and acrylics remain the most commonly used materials in prosthetics today. As research in this field progresses, development of new variations and fabrication techniques based on these synthetic polymers will lead to even better and more robust soft tissue prosthetics, with improved life-like aesthetics and lower cost manufacturing.

## Introduction

Congenital defects, trauma, or cancer often causes loss or disfigurement of tissue leading to distress and impairment for millions worldwide, significantly affecting their social, economic and psychological health (Tagkalakis and Demiri, 2009). The impact of physical disfigurement extends to the individuals’ body image, their perception of their physical self (Galpin, 1996; Tagkalakis and Demiri, 2009). In addition, disfigurement often leads to discrimination, bullying and less opportunities for the affected individual to participate fully in their society. Prosthetic devices have long been used to restore aesthetics and function to individuals with soft tissue disfigurement. Advances in materials and fabrication techniques over the centuries has enabled improvements in the capabilities of prostheses, particularly with respect to their aesthetics, attachment, function, cost and robustness.

Polymers are now used extensively in modern external prosthetics, having replaced many of the primary and natural materials that were available prior to their advent. The advantages of polymers extend to their ability to more realistically mimic native tissue both esthetically and functionally, as well as providing excellent safety, effectiveness, robustness and accessibility. Their application in prosthetics has also been extensively studied and the discovery of new prostheses and processing methods has led to radical shifts in many areas of prosthetic design. In some cases, this has led to significant advances in the realism and capability of prostheses with positive impacts on the lives of millions of people worldwide. Table 1 summarizes the mechanical and manufacturing properties of the polymers used in soft tissue prosthetics in modern times; some of which have been discontinued, many still in common use, and others still emerging.

**TABLE 1:** Properties of polymers historically used in soft tissue prostheses.

Polymer	Polymer repeat structure	Processing methods	Hardness (shore A)/tensile strength (MPa)	Pigmentation	Examples used in prosthetics
**Silicone: room temperature vulcanizing**					
One-part condensation		Painted onto surface as sealants, adhesives, and external colorants	28 – 35/2.0 – 3.3	Intrinsic colorants incorporated for application	Medical Adhesive Type A
Two-part condensation	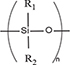	Simple casting	38 – 43/2.7 – 4.2	Intrinsic colorants incorporated prior to cure and extrinsic details added.	Discontinued usage
Two-part addition		Simple casting, 3D printing in development	25 – 32/4.8 – 5.0	Intrinsic colorants incorporated prior to cure and extrinsic details	A-2186, A-2186F, MDX4-4120
**Silicone: High temperature vulcanizing**					
Peroxide curing		Injection molding	25 – 75/5.9 – 6.9	Milling required for intrinsic colorants and extrinsic details added	Discontinued usage
Addition curing	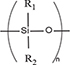	Press and injection molding	20 – 80/9.3	Milling required for intrinsic colorants and extrinsic details added	Q7-4720, Q7-4735, Q7-4750, Q7-4765, and Q7-4780
Liquid silicone rubber		Injection molding	24/8.4	Intrinsic colorants incorporated prior to cure and extrinsic details added	MED-4920 (NuSil)
Poly(methyl methacrylate)/PMMA/acrylic resin	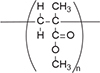	Simple casting with flexible molds, 3D printable	96 (Shore D)/ 68.3	Intrinsic colorants incorporated prior to cure and extrinsic details added.	Scleral acrylic resin (Factor II Inc.)
Polyvinyl chloride	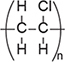	Simple casting with metal molds	53/4.0	Intrinsic colorants incorporated prior to cure and extrinsic details added.	RSL Steeper
Polyurethane	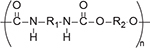	Simple casting as solid or foam, 3D printable	45/4.14 – 7.52	Intrinsic colorants incorporated prior to cure and extrinsic details added.	
Chlorinated polyethylene	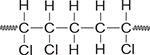	Thermoplastic material that is cast in layers, 3D printable	29/1.28	Milling required for intrinsic colorants and extrinsic details added	Tyrin CM0136

In this part of this two-part review, part B, we detailed the chemistry of common synthetic polymers in prosthetics, particularly their fundamental chemistry, synthesis, materials properties, fabrication and material degradation. In part A of this review, we discussed literature around the history of prosthetic materials, their desirable properties, some example applications to different tissues, and traditional and advanced manufacturing approaches to producing personalized soft tissue prosthetics. As stated in Section 1.2 of Part A, to mimic soft tissue a material should have a hardness between 25 and 35 Shore A (Shore hardness index), have a tensile strength of 6.9 to 13.8 MPa, be colorless and be easily intrinsically and extrinsically colored (Lewis and Castleberry, 1980). Simple processing methods are desired, such as simple casting methods with low cost molds or 3D printing.

We begin with a highly detailed description of the use and chemistry of silicone (polydimethylsiloxane), the most widely used polymer for mimicing soft tissues; including the usage and properties of the different types of silicones applied to prosthetics over its long history in this industry as well as current developments being made to lengthen the lifespan of silicone prostheses (Aziz et al., 2003a, b; Goiato et al., 2010a; Hatamleh and Watts, 2010c; Montgomery and Kiat-Amnuay, 2010). While silicone has predominantly replaced the more rigid acrylic resin in prosthetics, this important polymer was a forerunner in the domination of polymers in the prosthetic industry and still finds use in the fabrication of occular prostheses and as a substructure for weaker silicone prostheses (Chalian and Phillips, 1974; Craig et al., 1980; Artopoulou et al., 2006; Callaghan et al., 2006; Raizada and Rani, 2007; Goiato et al., 2014). Silicones have also nearly completely replaced the use of vinyl polymers in the fabrication of prostheses due to the improved color integrity and realistic feel. We also discuss literature around the degredation of silicone, particularly in the context of soft tissue prosthetics. However, before the development of stronger silicones, vinyls were the most favored prosthetic material for their high tear strength and softer feel when compared with rigid materials such as acrylic resin (Gearhart, 1970; Kenworthy and Small, 1974; Yu et al., 1983; Carroll and Fyfe, 2004; Smit et al., 2014). Polyurethanes have also been used as a prosthetic material, both as a bulk material and as a foam. However, they have not seen as wide spread use as silicone due to the difficulties of fabrication inherent with working with polyurethanes and tendancy toward yellow discoloration with aging (Chalian and Phillips, 1974; Goldberg et al., 1978; Craig et al., 1980). Chlorinated polyethylene, a newer prosthetic material has struggled to enter into common use since its introduction by the National Institute of Dental Research. Despite the potential of new 3D printable elastomeric materials (Çötert, 2015), silicone remains the material of choice for soft-tissue prostheses either due to ease of use or personal biases (Kiat-amnuay et al., 2010). The structure and properties of these polymers are summarized in Table 1.

In modern soft-tissue prosthetics polymers are widely used to restore aesthetics for conditions involving the ear (Ross et al., 2018), face (Fantini et al., 2013), eye (Alam et al., 2017), breast (Cancer Australia, 2019) and hand (Kaira and Dabral, 2014). These prosthetics are often hand-crafted by skilled prosthetists and tailored to the individual anatomy of each patient. Typically, physical casts are taken of the patient’s anatomy which are then used to produce molds into which the polymer is added for curing. More recent approaches involve the use of 3D scanning of the patient followed by computer modeling of the desired mold or prosthetic. Often, given the complexity of some prosthetics, reinforcement is required and included into the prosthetic. The following sections detail the chemistry of polymers used in prosthetics of the ear, face, eye, breast and hand. In part A of this review, the desired properties of polymeric materials used in soft-tissue prosthetics are discussed. We also discuss different approaches that have been used to address the need for realistic and robust prostheses.

## Silicone

Silicone, or silicone elastomer, typically refers to polydimethylsiloxane (PDMS). This popular polymer now has vast uses in a wide variety of industries from personal care to the automotive industry (Aziz et al., 2003b; Bellamy et al., 2003; Curtis and Colas, 2004; Hatamleh and Watts, 2010c). The first silicones were introduced in 1946 (Chalian and Phillips, 1974) and began being used in maxillofacial prosthetics in the 1960s (Barnhart, 1960). Today, it is the most widely used material in maxillofacial prosthetics, favored for its flexibility, heat resistance, transparency, and biocompatibility despite its inability to be modified or repaired (Aziz et al., 2003a, b; Goiato et al., 2010a; Hatamleh and Watts, 2010c; Montgomery and Kiat-Amnuay, 2010).

### Chemistry of Silicone and Prosthetic Fabrication

Silicone is produced when water is added to dimethyldichlorosilane, a compound formed by the reaction of silicon and methyl chloride. The resulting fluid polymer can then be cross-linked to form a solid. As shown in Table 1, the unique properties of silicone are a result of its chemical structure which is composed of an inorganic backbone of alternating silicon and oxygen atoms (siloxane structure) to which organic side groups, typically methyl (CH_3_), propyl ((C_3_H_7_) or phenyl (C_6_H_11_) groups, are bonded (Chalian and Phillips, 1974; Curtis and Colas, 2004; Lorenz and Kandelbauer, 2014). Comparison of the siloxane (Si-O) structure (Curtis and Colas, 2004; Lorenz and Kandelbauer, 2014) with the carbon backbone of organic polymers illustrates why silicones have such unique physical properties. The siloxane structure is strengthened by being composed solely of single bonds (saturation) as well as the high covalent bond energy between silicon and oxygen atoms (Curtis and Colas, 2004; Lorenz and Kandelbauer, 2014). The element silicon is also less electromagnetic and larger than carbon, allowing for greater flexibility (Curtis and Colas, 2004; Lorenz and Kandelbauer, 2014). This unique chemical structure gives silicone the advantages that make it a popular prosthetic material.

**FIGURE 1:**
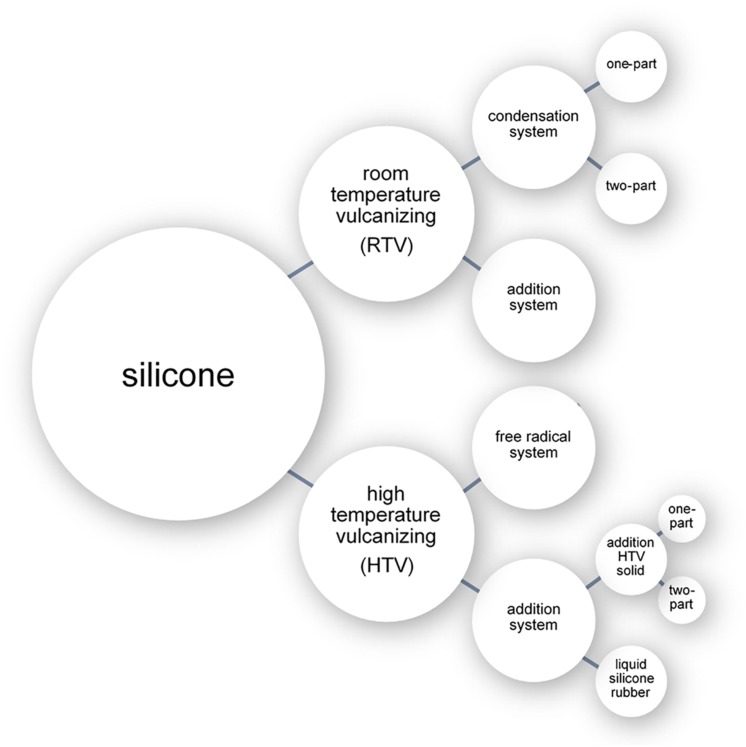
Diagram of the different classes of silicone.

The three chemical processes with which liquid silicone can be cross-linked are: free radical polymerization (peroxide curing), condensation polymerization, and addition polymerization (Figure 1; Colas, 2005; Andriot et al., 2009). Cross-linking processes can be broadly separated into room temperature approaches (room temperature vulcanizing systems or RTV) or elevated temperature approaches (high temperature vulcanizing systems or HTV) (Figure 1). While numerous silicone cross-linking approaches have been commonly used in prosthetic fabrication throughout the years, addition polymerization at room temperature has become the most common method due to its simplicity (Montgomery and Kiat-Amnuay, 2010).

#### Room Temperature Vulcanizing Silicone

Room temperature vulcanising (RTV) systems involve crosslinking by either condensation or addition polymerization using a catalyst and crosslinking agent. As the reaction occurs at room temperature, low-cost plaster and gypsum (dental stone) (Chalian and Phillips, 1974) can be used in the fabrication of the mold into which the silicone can be cured. The condensation polymerization systems are available as either a one-part (classified as RTV-1) or two-part (classified as RTV-2) system (Jerschow, 2001). Addition polymerization systems occur only as RTV-2 systems (Jerschow, 2001).

Condensation polymerization only occurs as room temperature vulcanizing systems with an organotin catalyst (e.g., stannous octoate) and crosslinker (e.g., methyl triacetoxy silane) (Jerschow, 2001; Lai et al., 2002; Aziz et al., 2003b; Curtis and Colas, 2004; Colas, 2005; Lorenz and Kandelbauer, 2014). Crosslinking begins as functional groups on the crosslinker become hydrolyzed to create silanols which trigger condensation and the release of a by-product (Jerschow, 2001; Lai et al., 2002; Curtis and Colas, 2004; Colas, 2005; Lorenz and Kandelbauer, 2014); the more common functional groups are acetoxy and alkyloxy groups, which polymerize to release acetic acid and methyl alcohol, respectively (Curtis and Colas, 2004; Lorenz and Kandelbauer, 2014). This reaction, if incomplete, can be reversed at temperatures exceeding 90°C (Jerschow, 2001; Curtis and Colas, 2004). Some of the disadvantages of the use of silicones cured through condensation polymerization in prosthetics include: long curing time, susceptibility of the material to degradation, low tear strength, low edge strength, and the formation of by-products which can lead to a porous structure, promoting sorption of liquids (Lai et al., 2002; Hulterström et al., 2008).

Room temperature vulcanising-1 condensation systems are commercially used as sealants and adhesives (Jerschow, 2001). As crosslinking begins immediately on contact with moisture in the air, they need to be stored in sealed cartridges. As moisture is required during its polymerization process, the practical cross-sectional thickness of the object being produced is limited, limiting their use in prosthetics (Lai et al., 2002; Curtis and Colas, 2004; Colas, 2005; Lorenz and Kandelbauer, 2014). Despite this, RTV-1 condensation systems have found use in prosthetics. An example of one such product is Medical Adhesive Type A (also called Silastic 891) (Dow Corning Company, Midland, MI, United States), which is solely used in external colorants on the surface of the prosthesis where it can be used in a thin layer to allow the passage of moisture throughout its cross-sectional thickness for complete polymerization (Lai et al., 2002). In a 1992 survey of American prosthetists (Andres et al., 1992), 35.2% of 88 respondents used Medical Adhesive Type A. In a more recent 2010 survey (Montgomery and Kiat-Amnuay, 2010), 39.5% of 43 respondents were still employing it for external detailing. RTV-1 condensation systems also have poor performance on a range of measures such as long time to complete polymerization, poor mechanical properties, and importantly, the creation of acetic acid (an irritant to skin) during production (Lai et al., 2002).

Crosslinking in RTV-2 condensation polymerization systems is initiated when the two components, a base and curing agent (catalyst), are combined without requiring the presence of moisture (Jerschow, 2001; Curtis and Colas, 2004; Colas, 2005; Lorenz and Kandelbauer, 2014). Commonly used silicone products using this curing process have been marketed in the past as Silastic 382 and Silastic 399 (Dow Corning Company). Silastic 382 was a viscous opaque white fluid base which was polymerized by a stannous octoate catalyst (Chalian and Phillips, 1974; Craig et al., 1980). Silastic 399 was viscous and non-flowing and required the addition of two different catalysts for polymerization (Craig et al., 1980). Up until the late 1980s, these materials were commonly used in the fabrication of implants and maxillofacial prostheses (Chalian and Phillips, 1974). However, concerns regarding their safety emerged in the 1980s (Lam and Hurry, 1992; Reisch, 1993; Cook et al., 1994; Wise, 2000; Curtis and Colas, 2004; Segal et al., 2012) and they were discontinued (Wise, 2000; Segal et al., 2012).

In room temperature vulcanizing addition polymerization systems (i.e., RTV platinum catalyzed silicones), unsaturated vinyl (–CH = CH2) terminated poly (siloxanes) are triggered by a platinum catalyst to react with silyl hydride (–SiH) groups and undergo polymerization (Lai and Hodges, 1999; Jerschow, 2001; Aziz et al., 2003b; Curtis and Colas, 2004; Colas, 2005). Though these are RTV systems, these silicones may be heat cured at temperatures up to 100°C to decrease curing time. One significant advantage to this polymerization approach is that shrinking does not occur as no by-product is created in this reaction (Jerschow, 2001; Curtis and Colas, 2004; Colas, 2005; Lorenz and Kandelbauer, 2014). The base component typically consists of dimethylsiloxane polymer, reinforced silica, and a platinum or rhodium catalyst (Lai and Hodges, 1999; Lai et al., 2002; Curtis and Colas, 2004). The curing agent consists of dimethylsiloxane polymer, an inhibitor, and a siloxane crosslinker (Lai and Hodges, 1999; Lai et al., 2002). In the context of their use in prosthetics, the disadvantages of addition polymerization include material hydrophobicity, selective adhesion, inability to be extrinsically stained, short working time and inhibition of curing by impurities (e.g., amines, sulfurous or other catalyst poisons) (Jerschow, 2001; Lai et al., 2002; Curtis and Colas, 2004; Lorenz and Kandelbauer, 2014).

Despite these limitations, the majority of maxillofacial prostheses are manufactured using RTV platinum catalyzed silicones (Montgomery and Kiat-Amnuay, 2010). The most popular being A-2186 (Factor II, Inc., Lakeside, AZ, United States), a clear two-part (10:1, base: catalyst) pourable silicone that was first introduced in 1986 (Montgomery and Kiat-Amnuay, 2010). A fast polymerization rate version was introduced in 1987 as A-2186F (Factor II, Inc.). A 1992 survey of 88 American prosthetists (Andres et al., 1992) found that 6.8% of respondents used A-2186 and a 2010 survey (Montgomery and Kiat-Amnuay, 2010) found that this had increased to 32.6% of 43 respondents. A-2186F, the faster polymerization rate version, did not appear in the 1992 survey, but was used by 20.9% of 2010 respondents. In the year 2000, A-2000 (Factor II, Inc.) was introduced as the first generation of 1:1 mixture platinum silicone, followed by A-2006 in 2006 (Factor II, Inc.) (Montgomery and Kiat-Amnuay, 2010); the 2010 survey found that these were used by 20.9 and 11.6% of respondents, respectively. MDX4-4210 (Dow Corning Company), another clear two-part (10:1, base: catalyst) pourable silicone, was first introduced to the maxillofacial industry in the 1970s and was most popular in the 1990s (Montgomery and Kiat-Amnuay, 2010). In the 1992 survey, MDX4-4210 was used by the majority (59.1%) of respondents, and was still used in the 2010 survey by 18.6% together with catalyst A-103 (Factor II, Inc.) and 16.3% together with Medical Adhesive Type A (Dow Corning Company).

#### High Temperature Vulcanizing Silicones

High temperature vulcanising (HTV) systems involve crosslinking by either free radical or addition polymerization. One of the advantages of high temperature vulcanizing silicones (between 100°C and 200°C) is the longer working time of approximately 30 min prior to polymerization. This, however, comes at a significantly increased cost over room temperature polymerization (Lorenz and Kandelbauer, 2014), and requires intense milling prior to polymerization for the incorporation of intrinsic pigments (Anusavice, 2013).

Free radical polymerization reactions (also known as peroxide-initiated reaction) are useful for producing high-consistency silicones (Curtis and Colas, 2004). By incorporating an organic peroxide to the silicone prior to heating, radicals involved in crosslinking are produced at high temperatures (Chalian and Phillips, 1974; Curtis and Colas, 2004; Colas, 2005; Lorenz and Kandelbauer, 2014). Typically, these silicones are catalyzed by dichlorobenzoyl peroxide (Craig et al., 1980) which is stable at room temperature and is activated at elevated temperatures (104–132°C); activating methylene groups that form ethylene crosslinks between chains of uncured polymer (Lorenz and Kandelbauer, 2014). The efficiency of this reaction is increased with the presence of vinyl groups in the polymer (Curtis and Colas, 2004; Colas, 2005). These silicones have high tear resistance and have excellent thermal stability and therefore ideal for prostheses where these properties are important. However, silicones cross-linked with radicals have low elasticity and therefore cannot be used in mobile regions, such as areas affected by jaw movement. Other disadvantages include opacity, yellowing after cure, odor during- and post-production, taste in the case of intra-oral prostheses, high friction (tacky) surface, release of peroxide split products, and possibility of peroxide residues which can create voids in the finished product as well as act as a catalyst for depolymerization at elevated temperatures (Jerschow, 2001; Curtis and Colas, 2004; Colas, 2005). Following high temperature polymerization, further processing may be applied to remove volatile peroxide residues (Jerschow, 2001; Curtis and Colas, 2004; Colas, 2005). Despite their tear resistance and thermal properties, the use of radical cross-linked silicones in prosthetics has been discontinued due to the availability of superior products, such as silicones produced by addition cure systems.

High temperature vulcanization through addition polymerization works similarly to RTV addition polymerization systems leading to silicones that are highly transparent with no yellowing, no odors, that are easy to demould, do not require post cure processing, and have high tear and tensile strength (Jerschow, 2001; Aziz et al., 2003b). These are available as either one-part systems (1K) with a shelf-life of 3–6 months or two-part (2K) systems with a shelf-life of 18 months when separated or 1–7 days once mixed (Jerschow, 2001; Dow Corning, 2006). Another feature of two-part systems is that flexibility can be tailored by altering the proportions of the two components.

Liquid silicone rubbers (LSR) are two-part addition curing compounds with consistency that can be tailored from pourable to pasty (Jerschow, 2001; Lorenz and Kandelbauer, 2014). The curing rate is also adjustable and occurs relatively slowly at room temperature due to the presence of both catalyst and inhibitor, and more rapidly at temperatures of 170°C to 200°C (Jerschow, 2001; Lorenz and Kandelbauer, 2014). The chemical structure of the cured material is similar to HTVs cross-linked with radicals, but the polymer chains are shorter (Lorenz and Kandelbauer, 2014). One example, MED-4920 (NuSil^TM^ Technology LLC, Carpinteria, CA, United States), is a 1:1 LSR that is used for prostheses; however, it is more commonly used in medical devices such as balloon catheters and tubing. It is translucent, moderately strong and can mimic soft tissue. Prior to curing, it is too viscous for pouring into a mold, but is suitable for injection molding (Aziz et al., 2003a, b; NuSil).

### Properties of Silicone in Prosthetics

Mechanical properties of silicone depend on three main factors; molecular weight, degree of crosslinking, and incorporation of fillers and pigments (Aziz et al., 2003b; Bellamy et al., 2003; Hatamleh and Watts, 2010c).

Molecular weight distribution has a direct effect on the strength and flexibility of the polymer. By blending long and short chains of the same polymer, a bimodal molecular weight distribution can be created (Aziz et al., 2003b). Shorter polymer chains (lower molecular weight) result in higher crosslinking which, in the case of silicone, results in a brittle inelastic material that does not mimic soft tissue. On the other hand, a low degree of crosslinking results in a highly elastic but weak material. It is therefore important to adjust the crosslinking density to balance between these two extremes to achieve a soft tissue prosthesis that also has a long service life (Aziz et al., 2003b; Bellamy et al., 2003).

Another approach to strengthen the mechanical properties of silicone and reduce its susceptibility to tearing is to incorporate filler. This is often referred to as extending, as it can lower the cost of the elastomer (Jerschow, 2001), thereby lowering the cost of the prosthesis. The filler works by dissipating energy during material deformation, allowing molecular chains to easily move past each other (Santawisuk et al., 2010; Zayed et al., 2014). It should be noted that, often the particles present in intrinsic coloring pigments can have a similar effect. The most common filler in silicone production is hydrophobic surface treated silica (SiO_2_ in the form of diatomaceous earth or ground quartz). This has been found to increase material hydrophobicity, increase strength, increase storage modulus, increase loss modulus, increase damping factor and decrease elasticity (Chalian and Phillips, 1974; Jerschow, 2001; Aziz et al., 2003b; Bellamy et al., 2003; Curtis and Colas, 2004; Santawisuk et al., 2010). Although these changes can increase the service life of the prosthesis, too much filler impacts tissue-like characteristics; leading to hardening and reduced comfort through reduced elasticity and decreased wettability.

In addition to silica, other materials have been explored for use as fillers. The incorporation of titanium, zinc, and cerium nano-oxides on strengthening silicone was investigated by Han et al. (2008). It was found that the addition of these nanoparticles in concentrations of 2.0 to 2.5%wt. increased the hardness, tear strength, tensile strength, and elongation of silicone at break. However, at higher concentrations of 3.0%wt., the nanoparticles were observed to have a tendency to agglomerate and thereby act as stress concentrating centers. This reduced the tear strength, tensile strength, and elongation of the silicone, effectively shortening the material’s service life. However, Zayed et al. (2014) found that silica (the most common filler) showed reduced agglomeration when incorporated as nano-sized particles (i.e., hydrophobic nano-SiO_2_ coated with silane coupling agent) instead of as typical macroparticles (Zayed et al., 2014), achieving significant increases in tear strength and elongation with a lower increase in hardness.

Other tested reinforcement materials include microspheres (Liu et al., 2013, 2015). These microspheres were fully enclosed, containing a light gas, thereby decreasing their overall weight (Liu et al., 2013). In one comprehensive study, Liu et al. (2013, 2015) tested microspheres of two materials for use as a reinforcement material: polymer microspheres 461 DET 40 d25 (acrylonitrile-vinylidene chloride methyl-methacrylate copolymer) and silica microspheres Permata MS 380E (SiO_2_). The polymer microspheres could be incorporated into the silicone without agglomeration at relatively low concentrations of 5 and 15%vol., however, at 30%vol., the microspheres tended to agglomerate creating locations for stress concentration and material failure (Liu et al., 2013). The polymer microsphere reinforced silicone demonstrated a similar wettability to normal silicone but had lower density, decreased thermal conductivity, improved shock absorption and increased tensile strength at concentrations of 5%vol., and increased elongation at break and increased hardness at concentrations of 5, 15, and 30% vol. (Liu et al., 2013). It was found, however, that the tear strength of the silicone decreased with increasing concentration of polymer microspheres, likely due to microsphere agglomeration (Liu et al., 2013). The study found that the silica microspheres, on the other hand, did not reportedly agglomerate but instead imbedded into the silicone matrix (Liu et al., 2013). The silica microsphere reinforced silicone showed improved shock absorption, increased tensile strength, increased elongation at break, and increased hardness with increasing concentration of silica microspheres. The overall results indicate that silicone containing silica microspheres had higher density and greater tensile strength and shock absorption, and similar tear strength compared with silicone containing polymer microspheres (Liu et al., 2015). This suggests that the inclusion of silica microspheres could potentially improve silicone prosthesis strength without compromising comfort and a realistic feel.

### Acrylic Substructures for Silicone in Prosthetics

**FIGURE 2:**
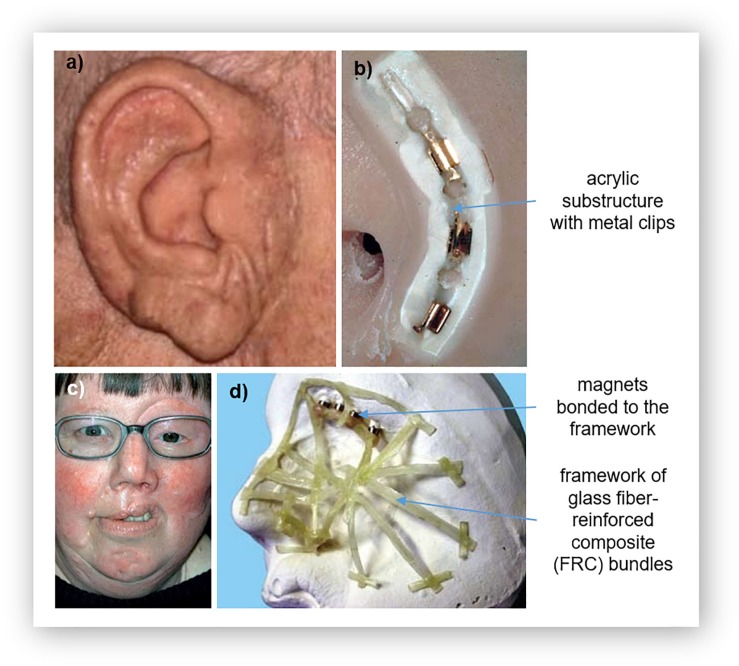
Acrylic substructures for silicone prostheses: **(a)** a prosthetic ear and its **(b)** substructure for clip attachment, and **(c)** a large facial prosthesis and its **(d)** glass fiber-reinforced composite (FCR) substructure for reinforcement. Reproduced with permission from Elsevier (Ciocca et al., 2007; Kurunmäki et al., 2008).

An important consideration for the use of silicones in prosthetics is the need to attach the prosthesis to the patient. Often, silicone prostheses are attached using osseointegrated implants along with a retentive structure that uses either bar clips (Figures 2a,b) or magnets (Figure 2c; Hatamleh and Watts, 2010a, c; Haddad et al., 2012; Yerci Kosor et al., 2015). The use of clips or magnets simplifies the routine of attachment of the prosthesis by providing guides. In many cases, these retentive structures are fabricated using acrylic resin (Hatamleh and Watts, 2010a, c; Haddad et al., 2012; Yerci Kosor et al., 2015). A few of examples are depicted in Figure 2, which shows the acrylic substructure on the attachment face of a silicone nose and ear and the substructure for a partial face prosthesis with magnetic attachment parts. To ensure adequate attachment of the prosthesis to the patient, it is therefore important that the silicone is suitably attached to the acrylic substructure.

Direct bonding between silicone and acrylic is difficult, as molecular adhesion also does not occur due to their different chemical structures (Hatamleh and Watts, 2010a; Haddad et al., 2012; Yerci Kosor et al., 2015). Adhesives have also been found to insufficient (Haddad et al., 2012). This challenge of enhancing the bond strength between silicone and acrylic resin has been shown to be overcome using primers that contain both an organic solvent and an adhesive agent (Hatamleh and Watts, 2010a, c; Haddad et al., 2012; Yerci Kosor et al., 2015). The primer acts as a chemical intermediate, reacting with both materials (Hatamleh and Watts, 2010a; Haddad et al., 2012); etching into the resin to enable the silicone to impregnate the surface of the resin by activating hydrogen bonds and covalent coupling. This causes swelling of the surface to increase wettability (Hatamleh and Watts, 2010a, c; Haddad et al., 2012; Yerci Kosor et al., 2015). While the adhesive acts on the silicone; the hydrophilic and hydrophobic groups react and bond with the functional groups of the silicone (Hatamleh and Watts, 2010a, c; Haddad et al., 2012; Yerci Kosor et al., 2015).

### Degradation of Silicone

Over time, all prostheses will undergo mechanical and chemical changes that limit their service life. Despite excellent durability, silicone eventually begins to look and feel unrealistic through color degradation, staining, weathering, changes to elasticity, and premature tearing. In addition, contact with the chemical environment of the skin secretions further degrades the polymer and also encourages microbial growth, leading to potential irritation and infection for the patient and microbial induced polymer degradation. Investigating ways to reduce this material degradation is important given the cost and complexities of producing many prostheses.

#### Time and Weathering

The greatest factor in the degradation of silicone’s mechanical properties is photo-oxidation. Photo-oxidation is usually attributed to environmental causes, particularly ultraviolet radiation; but also pollution, variations in temperature, and variations in humidity (Goiato et al., 2012b). Generally, this degradation mechanism can be described in three steps: initiation, propagation, and termination. Initiation occurs with the formation of free radicals (Eleni et al., 2009a, b, d, 2011b, 2011c; Rabek, 2012). For silicone, the inorganic backbone is highly resistive to irradiation due to the very high energy needed to cleave S-O bonds (Stathi et al., 2010). Therefore, the silyl radicals are often formed through cleaving of the methyl side groups (Rabek, 2012). During propagation, silyl radicals react with oxygen to produce polymer oxy radicals, peroxy radicals, and secondary polymer radicals, resulting in chain scissions (Eleni et al., 2009a, b, d, 2011b, 2011c; Rabek, 2012; Al-Harbi et al., 2015). Termination occurs when radicals react with each other, often creating crosslinks between the chains (Eleni et al., 2009a, b, d, 2011b, 2011c; Rabek, 2012; Al-Harbi et al., 2015). Initiation, propagation, and termination all occur simultaneously; with chain scission and crosslinking continuously occurring.

Another degradation mechanism for silicone is continual crosslinking that occurs over time. This has been seen in several nuclear magnetic resonance and infrared spectroscopy studies (Eleni et al., 2009d, 2011b, 2011c; Stathi et al., 2010; Hatamleh et al., 2011; Al-Harbi et al., 2015) and results in an increase in hardness, glass transition temperature, elastic modulus, and viscoelasticity; and a decrease in tear strength, maximum stress, and maximum strain. These changes, which significantly affect the feel of the prosthesis and lead to tearing, result from increases in the density of the structural network of the silicone as bonds continue to form between chains (Eleni et al., 2009b, d, 2011b, 2011c; Stathi et al., 2010; Hatamleh et al., 2011; Goiato et al., 2012b; Al-Harbi et al., 2015). This continued polymerization also occurs in the absence of environmental factors such as ultraviolet radiation. Silicone specimens stored in a dark room change their mechanical properties over time (at a rate lower than specimens exposed to weathering) due, in part, to continued polymerization well beyond the recommended time of curing (Guiotti et al., 2010; Polyzois et al., 2011).

In addition to mechanical changes, continued polymerization and photo-oxidation leads to unwanted color changes (Mancuso et al., 2009; dos Santos et al., 2010, 2011; Hatamleh and Watts, 2010b; Stathi et al., 2010). Both unpigmented and pigmented silicone undergoes accelerated color changes due to weathering, owing to enhanced crosslinking in the presence of UV radiation, air pollutants, temperature changes, and moisture (Hatamleh and Watts, 2010b; Stathi et al., 2010). However, the use of pigments has been shown to increase the rate of discoloration (Mancuso et al., 2009; dos Santos et al., 2010; Hatamleh and Watts, 2010b; Stathi et al., 2010; Al-Harbi et al., 2015); with organic pigments more susceptible than inorganic pigments to color changes (Mancuso et al., 2009; dos Santos et al., 2010). This color change is due to the migration of pigment particles within the polymer matrix (Mancuso et al., 2009; dos Santos et al., 2010); organic pigments are assumed to be larger than inorganic pigments, able to separate from the matrix more readily (Mancuso et al., 2009; dos Santos et al., 2010).

Another complication in the color degradation of silicone prostheses is that pigments of the same type (and manufacturer), but of different colors vary in their susceptibility to color change (Eleni et al., 2007, 2009a; Han et al., 2010; Stathi et al., 2010). This means that different prostheses made to match the skin of two different individuals may discolor at different rates.

In an attempt to maintain the aesthetic appearance of prostheses and lengthen their service lives, methods for reducing and preventing color change have been investigated (Han et al., 2010, 2013; dos Santos et al., 2011). One approach is through the incorporation of additives to decrease the translucency of the silicone. dos Santos et al. (2011), for example, found that barium sulfate (0.2wt%) prevented color change in unpigmented silicone, silicone pigmented with inorganic pigments, and silicone pigmented with functional pigments. This additive also has the advantage of strongly associating within the silicone matrix, thereby staying within the silicone and not greatly effecting the material hardness (Goiato et al., 2010a).

Han et al. (2010) tested titanium dioxide nanoparticles for inhibiting color change, finding that the addition of titanium dioxide nanoparticles can inhibit color change in silicone specimens with organic pigments. Furthermore, Wang et al. (2014) found that the addition of titanium dioxide has the added benefit of increasing tensile strength, increasing elongation at break, improving tear strength, and improving anti-thermal aging properties; with the disadvantage of increased hardness. This hardening effect of opacifiers, however, has been found to decrease following disinfection with neutral soap or effervescent (Goiato et al., 2010a). Both the addition of barium sulfate and titanium dioxide to silicone cause significantly higher dimensional changes during disinfection (Goiato et al., 2010a; Haddad et al., 2011). Furthermore, Han et al. (2013) found that commonly used opacifiers inhibited color changes in silicone in accelerated aging tests, but increased changes to the silicone’s mechanical properties.

#### Skin Secretions

During regular wear, prostheses are not only exposed to natural environmental conditions, but also the skin of the wearer. Polyzois et al. (2000); Eleni et al. (2011a), Hatamleh et al. (2011), and Al-Dharrab et al. (2013) performed comprehensive studies on the effects of simulated skin secretions such as acidic perspiration, alkaline perspiration, and sebum on the mechanical behavior of different commercial prosthetic silicones; Elastomer 42 (Technovent Ltd.), Techsil S25 (Technovent Ltd.), Cosmesil M511 (Technovent Ltd.), and Episil (Dreve-Dentamid GmbH). The results of these studies are summarized in Table 2. In short; acidic and alkaline perspiration is generally absorbed which weakens silicone while increasing elasticity and increasing hardness, and sebum interacts with the silicone surface with highly variable results depending on the type of silicone.

**TABLE 2:** Effect of skin secretions on different silicone products.

Material	Study	Types of testing	Acidic perspiration	Alkaline perspiration	Simulated sebum
Elastomer 42	Eleni et al., 2011a	Compression Hardness Absorption	↓ Maximum stress ↓ Maximum strain ↑ Elastic modulus ↓ Viscoelasticity parameter ↑ Hardness ↑↑ Weight	↓ Maximum stress ↓ Maximum strain ↑ Elastic modulus ↓ Viscoelasticity parameter ↑ Hardness ↑ Weight	↓ Maximum stress ↑ Maximum strain ↓ Elastic modulus ↑ Viscoelasticity parameter ↑ Hardness ↑↑ Weight
Techsil S25	Eleni et al., 2011a	Compression Hardness Absorption	↓ Maximum stress ↓ Maximum strain ↑ Elastic modulus ↑ Viscoelasticity parameter ↑ Hardness ↑↑ Weight	↓ Maximum stress ↓ Maximum strain ↑ Elastic modulus ↑ Viscoelasticity parameter ↑ Hardness ↑ Weight	↓ Maximum stress ↑ Maximum strain ↑ Elastic modulus ↓ Viscoelasticity parameter ↑ Hardness ↑↑ Weight
	Hatamleh et al., 2011	Tensile Tear Hardness	↓ Maximum stress ↓ Maximum strain ↑ Elastic modulus ↓ Tear strength ↑ Hardness	N/A	↓ Maximum stress ↓ Maximum strain = Elastic modulus ↓ Tear strength ↓ Hardness
Cosmesil M511	Eleni et al., 2011a	Compression Hardness Absorption	↓ Maximum stress ↓ Maximum strain ↑ Elastic modulus ↓ Viscoelasticity parameter ↑ Hardness ↑↑ Weight	↓ Maximum stress ↓ Maximum strain ↑ Elastic modulus ↓ Viscoelasticity parameter ↑ Hardness ↑ Weight	↓ Maximum stress ↑ Maximum strain ↑ Elastic modulus ↓ Viscoelasticity parameter ↑ Hardness ↓ Weight
	Al-Dharrab et al., 2013	Absorption	= Weight	= Weight	= Weight
Episil	Polyzois et al., 2000	Tensile Hardness Absorption	↑Maximum stress ↓ Maximum strain ↑↑ Elastic modulus ↑Tear strength ↑ Hardness ↑↑Weight	↑Maximum stress ↓ Maximum strain ↑ Elastic modulus ↓ Tear strength ↑ Hardness ↑ Weight	↑Maximum stress ↓ Maximum strain ↑ Elastic modulus ↑Tear strength ↓ Hardness ↓ Weight

These silicone property changes have been generally attributed to structural modifications in the distribution of the polymer chains (Hatamleh et al., 2011). In the case of silicone in simulated sebum; mechanical changes are attributed to interactions between fatty acids and the surface of the specimens (Polyzois et al., 2000; Eleni et al., 2011a; Hatamleh et al., 2011), breaking chain bonds (Hatamleh et al., 2011), increasing crosslinking density (Eleni et al., 2011a), and absorption of or secretion from silicone (Polyzois et al., 2000; Eleni et al., 2011a). Changes to silicone in simulated perspiration are attributed to the propagation of crosslinking reactions, creating a denser polymer network to increase elastic modulus and hardness (Polyzois et al., 2000; Eleni et al., 2011a; Hatamleh et al., 2011). Other changes include water absorption which leads to an increase in weight (Polyzois et al., 2000; Eleni et al., 2011a). However, the hydrophobic nature of silica fillers and vinyl functional silanes of some intrinsic pigments may prevent water absorption (Al-Dharrab et al., 2013). In particular, simulated acidic perspiration was found to have a possible catalytic effect on crosslinking, known as reversion; defined as the decomposition of junctions in the polymer network (Hatamleh et al., 2011).

Although silicone color changes due to simulated skin secretions were found to vary between different commercial silicones, they all showed a greater color change when placed in simulated sebum than in simulated perspiration except for Episil, which showed less color change in simulated sebum (Polyzois et al., 2000; Hatamleh and Watts, 2010b; Eleni et al., 2011a).

#### Microbial Growth

**FIGURE 4:**
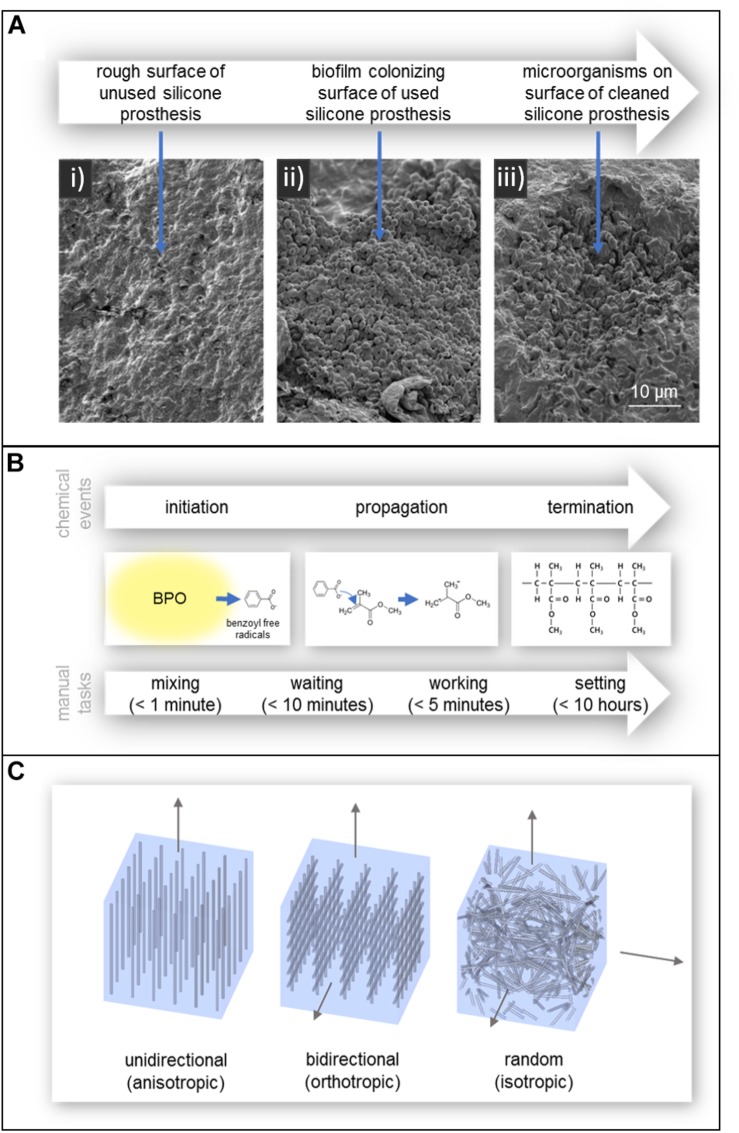
**(A)** SEM images of (i) the rough surface of an unused silicone prosthesis, (ii) a biofilm colonizing the surface of a used silicone prosthesis, and (iii) microorganism remaining embedded in the defects of the prosthesis after cleaning. Reproduced from *Taylor and Francis* (Ariani et al., 2012). **(B)** Polymerization of acrylic resin and the manual tasks associated with working with autopolymerising acrylic resin. Times are according to manufacturer Factor II, Incorporated (Product Information - Heat Cured Acrylics, 2010). **(C)** Fiber reinforcement of acrylic resin; (left) unidirectional, (center) bidirectional, and (right) randomly oriented.

In addition to affecting polymer degradation, contact with skin promotes microbial growth. This is known to adversely affect the mechanical properties and appearance of the prosthesis reducing its service life, as well as cause irritation and possibly infection on the skin of the patient. While silicone itself does not chemically promote the growth of microorganisms, the porosity and surface roughness of silicone allows the material to be colonized by a variety of commensal microorganisms which form biofilms and resist removal. This can be seen in the SEM images of Figure 4a (Goiato et al., 2010c; Rodger et al., 2010; Ariani et al., 2012). Additionally, the hydrophobic nature of silicone aids in microbial colonization and the tendency of silicone to accurately reproduce the surface of the molding material can lead to a rough surface environment ideal for microbial growth (Hulterström et al., 2008; Preoteasa et al., 2011; Ariani et al., 2012).

When biofilms form on silicone, microorganisms are able to penetrate into the silicone matrix and create bag-like defects and reduce the service life of the prosthesis (Rodger et al., 2010; Ariani et al., 2012). The two mechanisms behind this degradation of silicone are believed to be: mechanical degradation due to turgor pressure of blastospores and hyphae in the pores of the material, and chemical degradation due to the release of extracellular enzymes or free radicals (Rodger et al., 2010). Interestingly, a study by Rodger et al. (2010) found that an increase in filler content may aide to hinder the colonization of C. albicans, one of the more common commensal microorganisms.

#### Disinfection

The service life and quality of a prosthesis can generally be extended by regularly cleaning and disinfecting to remove skin secretions and microorganisms. However, the cleaning products and disinfectants themselves can also degrade the silicone. Several studies have investigated the degradative effects of different disinfection methods. These include studies on microwave disinfection, the use of effervescent tablets, 4% chlorhexidine gluconate solution, 1% sodium hypochlorite solution, neutral soap, and commercial disinfectants (Goiato et al., 2008, 2009, 2010a, 2010b, 2012b; Guiotti et al., 2010; Haddad et al., 2011; Hatamleh et al., 2011; Eleni et al., 2013a, b; Kotha et al., 2016).

The effect of storing silicone in sodium hypochlorite solution, neutral soap, and a commercial disinfectant on material hardness have been tested by Hatamleh et al. (2011) and Eleni et al. (2013a; 2013b). When measuring hardness with a durometer, they found that all three disinfection methods led to an overall decrease in material hardness (Eleni et al., 2013a). However, microindentation tests performed by the same group in a second study found that both neutral soap and the commercial disinfectant caused an increase in material hardness and elastic modulus (Eleni et al., 2013b). This apparent conflict between results was thought to be due to an overall absorption of solution into the silicone generally decreasing its bulk hardness, but extraction of surface compounds by neutral soap and the commercial disinfectant increasing hardness. In addition to changing the hardness of the silicone, storage in commercial disinfectant has also shown to significantly decrease tear strength (Hatamleh et al., 2011).

The effect of other disinfection techniques on the properties of silicone have also been investigated. Eleni et al.(2013a; 2013b), as previously discussed, investigated microwave disinfection by immersing silicone samples in water and microwaving for 3 min, 365 times, to simulate daily disinfection for 1 year. While the hardness appears to decrease by a small amount when measuring with a durometer (Eleni et al., 2013a), microindentation tests showed an increase in hardness (Eleni et al., 2013b). Additionally, Kotha et al. (2016) demonstrated that longer duration microwave disinfection at 8 min damaged the surface of silicone and reduced tensile strength.

Less frequent disinfecting appears to reduce the negative effects of disinfectants. Several studies using the disinfectants chlorhexidine, effervescent method, and neutral soap, did not see any significant change in mechanical properties or dimension of silicone without additives (Goiato et al., 2008, 2009, 2010a, 2010b, 2012b; Haddad et al., 2011). However, specimens with additives including ceramic pigments, make-up, or titanium dioxide opacifiers (used to match the appearance of patient’s tissue); showed changes in mechanical properties even with the reduced disinfection regime. Additionally, the effect of different methods of prosthetic silicone disinfection vary between different additives, such as pigments and opacifiers (Goiato et al., 2010a; Guiotti et al., 2010).

## Acrylic Resin

**FIGURE 3:**
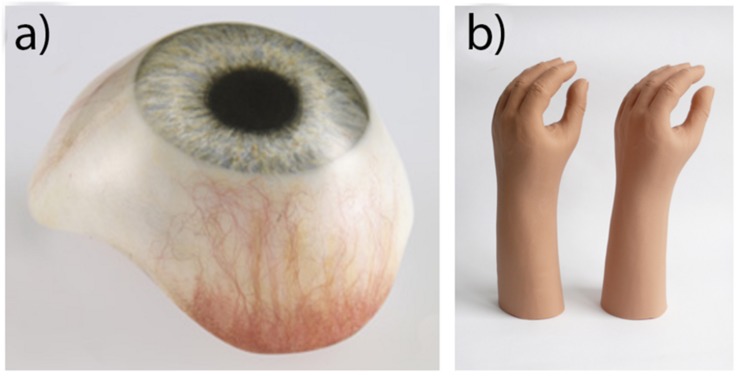
**(a)** Acrylic prosthetic eye. Reproduced with permission from Erickson Labs Northwest (Northwest_Eye_Design, 2019). **(b)** PVC glove (left) and silicone glove (right), illustrating an equivalent aesthetic appearance. Reproduced with permission from Sage (Smit et al., 2014).

Acrylic resin typically refers to the polymer poly (methyl methacrylate; PMMA). It is a clear rigid polymer mostly used as a dental base material, but also used in the fabrication of prostheses. It also has important application in prosthetic substructures for softer materials like silicone (Chalian and Phillips, 1974; Craig et al., 1980; Callaghan et al., 2006; Goiato et al., 2014). In 1944, during World War II when there was a shortage of glass, the United States Naval Dental and Medical School developed a technique to fabricate prosthetic eyes using acrylic resin (Artopoulou et al., 2006; Patil et al., 2008). The acrylic prosthetic eyes were found superior to glass; being lightweight, easy to fit and adjust, stronger than glass, translucent, easily fabricated, able to be intrinsically and extrinsically colored, and inert to socket secretions (Artopoulou et al., 2006; Raizada and Rani, 2007). As a result, acrylic resin replaced glass as the preferred material in the fabrication of prosthetic eyes like the one shown in Figure 3a. Unlike with silicone, there is no widely preferred products among acrylic resins, with most studies preferring the use of locally available dental resins with both (Bindhoo and Aruna, 2011; Cevik et al., 2012; Goiato et al., 2012a; Goyal et al., 2012; Ruiters et al., 2016; Shrivastava et al., 2013; Tomar et al., 2018), though many studies in India prefer dental acrylic resin from Dental Products of India (Mumbai, India) (Gupta and Padmanabhan, 2012; Veerareddy et al., 2012; Pruthi and Jain, 2013; Thirunavukkarasu et al., 2014; Tripuraneni et al., 2015; Shankaran et al., 2016; Shetty et al., 2016).

### Chemistry of Acrylic Resin and Fabrication in Prosthetics

Acrylic resin is composed of units of methyl methacrylate (MMA), an ester of methacrylic acid (Callaghan et al., 2006). MMA consists of a backbone of two carbon atoms connected by a double bond; one of which is covalently bonded to two hydrogen atoms and the other is covalently bonded to a methyl and an acrylic group (Callaghan et al., 2006).

As the polymerization process of pure MMA monomer is quite slow, taking hours to days to cure, a more efficient method of polymerization was needed for many applications, including prosthetics. In 1936, Walter Bauer developed a solution that efficiently fabricates acrylic resin. His technique is still in use today in a variety of industries including prosthetics and dentistry (Ratner et al., 2004; Callaghan et al., 2006; Pan et al., 2013). The method works by way of a two-component system; a powder component consisting of pre-polymerized solid acrylic resin beads 10 to 150 μm in diameter, and a liquid component consisting of MMA monomer. These two components are then combined to form a dough-like material (Dumitriu, 2001; Ratner et al., 2004; Callaghan et al., 2006; Goiato et al., 2014). One of the major advantages of the two-component system in prosthetic fabrication is the ease of manipulation due to the doughy consistency of the mixed material, minimization of the heat produced (as MMA polymerization is a highly exothermic reaction), and minimization of volumetric shrinkage that occurs during MMA polymerization (therefore maintaining shape and accuracy) (Ratner et al., 2004; Callaghan et al., 2006).

The mixing of these two components can be achieved in three ways; by hand mixing alone, hand mixing followed by centrifugation, and mixing in an evacuated device (vacuum mixing) (Callaghan et al., 2006). Hand mixing is performed by combining the components in an open bowl and mixing with a spatula, creating pores in the mixture. As the mixing continues, this porosity increases. One of the drawbacks with this method is that the pores can create sites of stress concentration and also microbial growth. Centrifugation after hand mixing attempts to remove these pores by removing air from the mixture (Callaghan et al., 2006), during which MMA is often chilled to decrease the rate of polymerization, reduce mixture viscosity, and allow trapped air to evacuate (Callaghan et al., 2006). Mixing in a vacuum, while most effective at decreasing porosity and preventing MMA monomer residue, can lead to excessive shrinkage and the formation of cracks, causing inaccuracies and leaving the material prone to failure (Callaghan et al., 2006).

One of the challenges with the two component mixing approach is that pure MMA polymerizes readily if exposed to light or heat (Dumitriu, 2001), such that the liquid component requires a stabilizer (e.g., hydroquinone) to absorb any free radicals that may appear (Dumitriu, 2001; Callaghan et al., 2006).

As shown in Figure 4b, the polymerization process occurs in three chemical stages; initiation, propagation, and termination (Callaghan et al., 2006). In the initiation stage, benzoyl peroxide (BPO), incorporated into the powder component, is activated and decomposes into benzoyl free radicals (Ratner et al., 2004; Callaghan et al., 2006). This can occur in several ways; heat by water bath, heat by microwave radiation, incorporation of an activator (autopolymerisation/self-curing), or light (Goiato et al., 2014). The benzoyl free radicals then react with MMA monomer in the propagation stage, breaking the double bond between two carbon atoms. The MMA monomer then becomes a free radical which continues to react with another MMA monomer or attach to prepolymerised resin and the process repeats (Callaghan et al., 2006). This process ends in the termination stage, where propagation stops by chain coupling. However, not all the MMA monomers become polymerized as the curing of the polymer makes monomer diffusion more difficult. As a result, some residual monomer remains in the final polymer (Callaghan et al., 2006).

One polymerization approach, heat polymerization, involves heating the uncured resin to just above 60°C, at which BPO is activated. The advantage of this approach in prosthetics is that cheaper dental stone (gypsum) molds can be used, lowering the overall cost of the prosthesis (Chalian and Phillips, 1974; Craig et al., 1980; Ratner et al., 2004). The application of heat is achieved using a heated water bath or microwave. Of these, microwave curing has been shown to lead to a more uniform distribution of polymerization throughout the matrix and therefore, less shape distortion (Fernandes et al., 2009b).

Autopolymerisation or self-curing relies on the incorporation of chemical agents, as activators, to initiate polymerization. For BPO, this is typically an amine activator (N, N-dimethyl-p-toluidine or DMPT) (Dumitriu, 2001). Acrylic resin cured via the autopolymerisation approach releases more residual monomer than heat-polymerized resin. This leads to a greater color instability, reducing the aesthetic appearance of the final prosthesis. Another drawback is the possibility of higher cytotoxicity levels which is highly undesirable for a prosthesis regularly in contact with a person’s skin or mucosal cavities such as the mouth and eye socket (de Andrade Lima Chaves et al., 2012; Goiato et al., 2014).

In the context of prosthetic production, polymerization of acrylic resin can alternatively be divided into four stages according to the associated manual tasks; mixing, waiting, working, and hardening/setting periods, as shown in Figure 4b (Callaghan et al., 2006).

The mixing stage involves dissolving the acrylic resin powder into the MMA monomer (Callaghan et al., 2006). The mixture becomes viscous, resulting in a tacky and paste-like consistency (Ratner et al., 2004; Callaghan et al., 2006). During the waiting period, viscosity increases until the mixture becomes doughy (Ratner et al., 2004; Callaghan et al., 2006). The working period begins when the dough is no longer tacky and can be worked and molded (Callaghan et al., 2006). In the final hardening/setting stage for autopolymerising resin, polymerization will continue with increasing viscosity and the production of heat (Callaghan et al., 2006). The final hardening stage for heat-cured resin requires them to be placed in an oven to cure. Following polymerization, prosthetic finishing includes polishing and heat treatment to smooth any rough surfaces and remove any residual monomer.

### Properties of Acrylic Resin

Acrylic resin is a hard rigid material suitable as a prosthesis for rigid areas of the body such as the eye, and is also used as a reinforcing material in composite prostheses (dos Santos et al., 2012). Although highly suited to these applications, limitations include the formation of pores (or voids), volumetric shrinkage, incomplete polymerization, and tendency to fracture (Pan et al., 2013).

The mechanism behind shrinkage relates to the differing densities of MMA monomer (0.943 g/ml) and polymerized acrylic resin (120 g/ml). This shrinkage is minimized by the incorporation of acrylic resin powder; the typical mixing ratio being 1:3 (vol./vol.) MMA liquid to acrylic resin powder, respectively. This change in volume during polymerization leads to incomplete polymerization and pores may be introduced into the final product. Although this shrinkage is usually below 7%, it is still problematic in prosthetic applications that require high accuracy (e.g., connecting osseointegrated implants) (Callaghan et al., 2006).

Pores can be introduced through air dissolved in powder particles, aeration during mixing, incomplete fusion of acrylic resin beads with MMA monomer, and evaporation of MMA monomer at temperatures greater than 100°C (Callaghan et al., 2006). Pores lead to incomplete polymerization; adversely affecting the mechanical properties of acrylic resin, including mechanical strength, surface roughness and hardness (Callaghan et al., 2006; Fernandes et al., 2009a, 2010). Pores which exceed the critical size of 70 μm also act as sites for stress concentration (Callaghan et al., 2006). They also provide locations for microbial growth which can alter the material color, degrade the mechanical properties of the acrylic resin and potentially infect the patient (Fernandes et al., 2010).

Another issue with incomplete polymerization is the presence of toxic chemical residues which are undesirable when used in contact with skin and mucosal cavities, such as the mouth and eye socket (Fernandes et al., 2009a; Goiato et al., 2014). These include formaldehyde, methacrylic acid, benzoic acid, dibutyl phthalate, phenyl benzoate, phenyl salicylate, and MMA monomer (Goiato et al., 2014). For intra-oral applications, polishing is required to reduce gingival (gum) inflammation. Acrylic resin can be exposed to heat treatments or water immersion for at least 24 h to reduce the quantity of residues (Siqueira Gonçalves et al., 2008; Ata and Yavuzyılmaz, 2009; Bural et al., 2011; Saravi et al., 2012; Goiato et al., 2014). Additionally, the replacement of 10wt% of MMA monomer with dimethyl itaconate (DMI) and di-n-butyl itaconate (DBI) can reduce water sorption and residual monomer content (Spasojevic et al., 2015). In the context of intra-oral applications, this replacement would reduce gingival inflammation but decrease storage modulus, ultimate tensile strength, and impact fracture resistance; resulting in a prosthetic material more susceptible to fracture.

### Degradation of Acrylic Resin

Like other materials used in prosthetics, the aesthetic and mechanical characteristics of acrylic resin degrades during use. This can occur from exposure to liquids, mechanical forces, thermal changes, and exposure to ultraviolet radiation. These changes negatively affect the aesthetic appearance of the prosthesis by changing the material color, and can degrade the material’s mechanical properties leading to a greater tendency to deform or to fracture.

Acrylic resin is known to absorb water due to its polar nature (Bettencourt et al., 2010) in a process known as imbibition; the absorbed water separates the polymer chains, causing expansion of the resin and reduces flexural strength which, again, results in a greater tendency to fracture (Ekstrand et al., 1987; Fernandes et al., 2009b; Goiato et al., 2012a). Water sorption in an acrylic prosthesis also causes the diffusion of unbound/uncured monomers and/or additives from the polymer matrix (Bettencourt et al., 2010); wet artificial weathering and exposure to saliva have been found to increase microhardness due to the elimination of unpolymerized monomer on the resin surface (Fernandes et al., 2009a; Bettencourt et al., 2010). If in contact with saliva as in intra-oral prostheses, the esterases present in saliva encourage the esterification of methacrylates in the resin (Bettencourt et al., 2010). As such, contact with water or saliva may have a plasticizing effect by creating more distance between polymer chains (Ekstrand et al., 1987; Fernandes et al., 2009b; Bettencourt et al., 2010; Goiato et al., 2012a) (susceptible to deformation) or make the resin more rigid (susceptible to fracture), depending on the plasticizing effect of the additives that are leached out (Bettencourt et al., 2010).

Another source of acrylic polymer degradation in prosthetics is through applied mechanical forces, particularly weak repetitive loads such as facial movements, leading to material fatigue in the polymer matrix (Bettencourt et al., 2010). These loads, combined with voids and residual stresses already present in the matrix, encourage the initiation and propagation of cracks leading to increased water absorption, and in turn, fracture (Bettencourt et al., 2010). Internal and surface stresses can also be created by thermal changes combined with differences in linear coefficients of thermal expansion between the acrylic resin and any materials adhered within or outside of the acrylic resin (Bettencourt et al., 2010).

Color change in pigmented acrylic resin is primarily due to degradation of the pigments themselves. However, colorless acrylic resin still discolors (yellow) with age (dos Santos et al., 2010), reducing the aesthetic appearance of the prosthesis. One of these sources of color degradation is ultraviolet degradation, which can be mitigated though the incorporation of inorganic nanoparticles to absorb and dissipate much of the ultraviolet light. Unfortunately, these particles can also negatively affect flexural strength of the material (Andreotti et al., 2014).

### Reinforcement of Acrylic Resin

In many prosthetic applications, the strength of some components must be enough to withstand high loads. To strengthen acrylic resin and lengthen the service life of prostheses, fibers of inorganic material (e.g., glass, carbon/graphite, and Kevlar) or high modulus polyethylene fibers may be added for reinforcement (Ekstrand et al., 1987; Vallittu, 1998, 1999; Uzun et al., 1999; Kanie et al., 2000, 2004; Chen et al., 2001; Kim and Watts, 2004; Narva et al., 2005; Pan et al., 2013). These fibers have been shown to increase properties such as impact strength and modulus of elasticity (Ekstrand et al., 1987; Uzun et al., 1999; Vallittu, 1999; Narva et al., 2005). There are several factors affecting the strength of such composites; including fiber orientation, fiber concentration, adhesion between the fibers and resin, and the fibers and resin themselves (Meriç and Ruyter, 2008). Limitations still exist in the ability to successfully reinforce acrylic, such as achieving fiber homogeneity and the desired fiber orientations, limited ability to sufficiently saturate the fibers in resin (leading to voids), and methods to avoid defects (Vallittu, 1999; Pan et al., 2013).

Unidirectional fiber reinforcement provides anisotropic mechanical properties (Meriç and Ruyter, 2008); strengthening and stiffening the material under load only along the direction of the fibers can be seen in Figure 4c (Uzun et al., 1999; Meriç and Ruyter, 2008). Bidirectional fiber reinforcement, such as woven glass fibers, provide enhanced orthotropic mechanical strengthening only along the surface of the fiber mesh, but much lower than with unidirectional fibers. Randomly oriented fibers provide isotropic (in all directions) material strengthening (Meriç and Ruyter, 2008).

Fiber density within the acrylic resin matrix significantly impacts the resulting composite material properties. A higher concentration of fibers within the matrix improves flexural characteristics (Chen et al., 2001; Meriç and Ruyter, 2008). Chen et al. (2001) found that impact strength increases with increased fiber concentration, however, a practical concentration limit of 3wt% was found, beyond which it became difficult to manipulate the material into the desired form. Another advantage of increasing fiber concentration is that it decreases the material’s ability to absorb water, thereby maintaining mechanical properties in wet environments of mucosal cavities such as the mouth and eye socket (Meriç and Ruyter, 2008). Chen et al. also found that longer fibers resulted in higher impact strength and that none of the fibers tested (polyester, Kevlar, and glass) significantly affected bending strength or surface hardness of the final reinforced acrylic (Chen et al., 2001).

Issues that must be considered when reinforcing acrylic with fibers are voids that may arise due to the insufficient saturation of fibers. These act as oxygen reserves which inhibit polymerization and increase the percentage of residual monomer in the final prosthesis (Vallittu, 1999). Further defects can also arise following fabrication of the prosthesis during everyday use from insufficient adhesion between the fiber reinforcer and resin, making the prosthesis more prone to failure. Considering this, glass has shown superior adhesion to acrylic resin when compared with polyethylene fibers (Vallittu, 1999; Meriç and Ruyter, 2008). Further improvements in glass/polymer adhesion can be gained by using techniques such as salinization or pre-treating these glass fibers with monomer, improving the wettability of the fibers during impregnation (Meriç and Ruyter, 2008; Meriç et al., 2008).

Achieving homogenous distribution of reinforcing fibers throughout the acrylic resin is mechanically challenging. Efforts to overcome this have been attempted by using pre-impregnation of reinforcing glass fibers with resin. The products Stick and StickNET (GC EUROPE, Leuven, Belgium) pre-impregnate continuous unidirectional glass fibers or woven glass fiber with porous resin, respectively (Vallittu, 1999). The voids in the porous resin allow monomers to penetrate into the existing resin matrix when combined with powder and liquid resin (Vallittu, 1999).

Failure to achieve optimal fiber reinforcement can result in stress concentration in the material, leading to fracture and to a reduction of tensile strength below suggested theoretical values (Ekstrand et al., 1987; Vallittu, 1998). Furthermore, the use of inorganic materials as reinforcements have shown to cause mucosal irritation and damage in areas such as the mouth and eye socket (Pan et al., 2013). Voids may also be produced due to poorly saturated fibers, encouraging microbial growth (Vallittu, 1999). Other issues extend to difficulties in achieving an aesthetic natural appearance when incorporating dark-colored fibers (i.e., carbon/graphite and Kevlar). This is not an issue, however, with glass fibers and polyethylene fibers which are almost invisible when incorporated into acrylic resin (Uzun et al., 1999).

## Vinyl Polymers

Plasticized polyvinyl chloride (PVC) was once the most widely used material in soft tissue prosthetics, and is still used today in the production of gloves for prosthetic hands like the one depicted in Figure 3b (Gearhart, 1970; Kenworthy and Small, 1974; Carroll and Fyfe, 2004; Smit et al., 2014). It was once favored over silicones due to its lower costs, higher tear strength and lighter weight (Yu et al., 1983; Carroll and Fyfe, 2004). However, in the 1970s, its use began to dwindle as new stronger RTV silicones were developed with a higher tear strength. The increased production of these newer silicones continued over the decade, reducing their cost (Gearhart, 1970). By the time of the 1992 survey of American prosthetists, only one in 88 respondents used PVC (Andres et al., 1992). Compared to PVC, prostheses made using RTV silicone are now easier to manufacture, contain better color integrity, and have more human skin-like characteristics in both appearance and feel.

### Chemistry of Vinyl Polymers and Fabrication in Prosthetics

**FIGURE 5:**
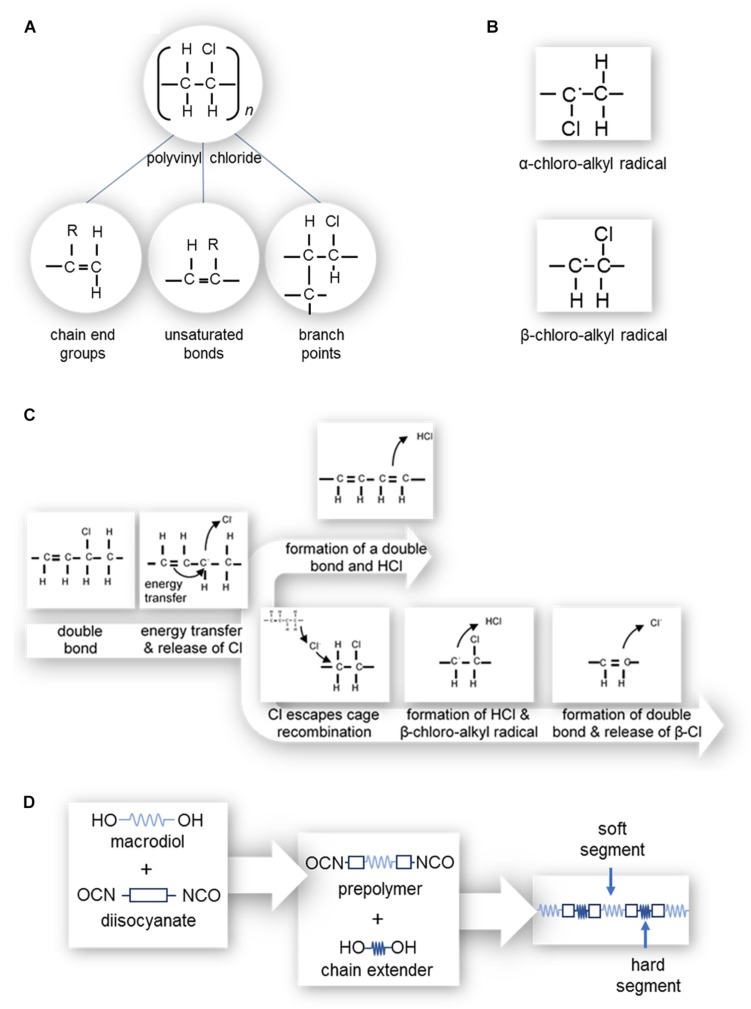
**(A)** Polyvinyl chloride structure and possible defects, where the R can be either a hydrogen or chlorine atom; (left) chain end groups with an unsaturated bond, (center) branch points, and (right) unsaturated bonds along the length of the polymer chain. **(B)** α-chloro-alkyl and β-chloro-alkyl free radicals. **(C)** Zip dehydrochlorination of PVC. **(D)** Polymerization of polyurethane.

An ideal PVC molecule would only contain single bonds of C-C, C-H, and C-Cl. However, defects as shown in Figure 5a tend to be present; such as unsaturated bonds (allylic chlorine), chain end groups, and branch points (i.e., tertiary-bonded chloride atoms and oxidized structures) (Shi et al., 2008; Singh and Sharma, 2008; Rabek, 2012). Unsaturated bonds (i.e., multiple bonds) enhance material degradation, discoloration and changes in mechanical properties. The degree of polymerization (i.e., number of monomeric units in a macromolecule) also impacts the number of defects present, producing locations susceptible to degradation. Shi et al. (2008) found that while PVC with a degree of polymerization of 800, 1000, or 1300 had small numbers of defects, PVC with a degree of polymerization of 3000 contained a larger number of pendant double bonds due to copolymerization with a crosslinking agent, hence a larger number of defects.

For many prosthetic applications, PVC plastisol resin is available for use. This PVC resin comprises a moderately viscous to putty-like suspension of solid polymer within a liquid plasticizer (Craig et al., 1980; Yu et al., 1983). The use of the plasticizer allows time for molding the prosthesis before PVC polymerization, but also results in shrinkage upon polymerization. When the resin is heated to 200°C using high temperature molds (Craig et al., 1980; Hutcheson and Udagama, 1980), the solid polymer swells and dissolves in the plasticizer. The mixture then becomes a gel as the temperature is lowered (Yu et al., 1983).

Plasticizers used for PVC production are usually from a group of chemicals called phthalates, particularly di-2-ethyl hexyl phthalate (DEHP). This plasticizer is usually incorporated with concentrations of at least 30wt% of PVC plastisols (Vedanarayanan and Fernandez, 1987; Heudorf et al., 2007). Phthalate plasticizers are commonly used and can be found in a range of everyday items such as building materials, household furnishings, clothing, cosmetics, pharmaceuticals, nutritional supplements, medical devices, dentures, toys, glow sticks, modeling clay, food packaging, automobiles, lubricants, waxes, cleaning materials and insecticides (Schettler, 2006; Heudorf et al., 2007).

### Properties of Vinyl Polymers

Pure PVC is a clear, hard and rigid plastic (Chalian and Phillips, 1974; Craig et al., 1980). It is tasteless, odorless, darkens and yellows when exposed to UV, can be intrinsically and extrinsically stained, is insoluble in inorganic solvents and soluble in aqueous solutions. Furthermore, it can be degraded by microorganisms found in septic systems, landfills, compost and soil by enzymatic processes (Chalian and Phillips, 1974; Craig et al., 1980). To enhance the softness and elasticity of PVC, plasticizers (phthalates) are added to produce PVC plastisol (Craig et al., 1980). This compound, however, is still stiffer in comparison with typical RTV silicones.

In general, prostheses made from plasticized PVC have a natural appearance with a texture similar in feel and pliability to skin. They also have a basic translucency similar to natural flesh and are relatively easily processed, easily colored intrinsically and extrinsically, easily cleaned, retain shape, and are fairly durable (Gearhart, 1970). One of the drawbacks of plasticized PVC for use in prosthetics is their limited tear resistance which is too low to allow for molding extremely thin edges, required to integrate esthetically with native tissue. Another disadvantage is that they are environmentally unstable; being susceptible to drying and cracking, tackiness, and color changes (Gearhart, 1970; May and Guerra, 1978).

The use of phthalates as plasticizers for PVC, which enable PVC to have human skin-like properties, has important potential biocompatibility implications. As there is almost no chemical bonding between phthalates and PVC, phthalates in prostheses can leach from PVC by saline, anticoagulant citrate dextrose (ACD) solution, plasma and blood (Vedanarayanan and Fernandez, 1987; Heudorf et al., 2007). Exposure to phthalates is achieved through ingestion, inhalation, and dermal exposure. This is, in part, due to their lipophilic nature which allows them to pass through the human bi-lipid cell membrane (Schettler, 2006). Phthalates have also been extensively investigated for many possible and significant toxic effects. These include carcinogenicity, disruption of the reticulo-endocrine systems (encouraging platelet aggregation), reduction of birth weight for fetuses exposed through their mother’s blood, shortening of anogenital distance in males, reduction of serum testosterone levels, and decrease of spermatocyte numbers (Vedanarayanan and Fernandez, 1987; Schettler, 2006; Heudorf et al., 2007). While DEHP, in particular, has been found to cause hepatocellular carcinoma and other hepatocellular effects in rodents, there is no evidence that there are carcinogenic effects within the human population (Heudorf et al., 2007). There is, however, evidence to suggest that DEHP may cause disruption of the reticulo-endocrine system (Heudorf et al., 2007).

In addition to plasticizer, the vinyl chloride monomer itself possesses toxic effects and is a known human carcinogen affecting the liver (angiosarcoma), brain, lungs, and hematopoietic and lymphopoietic systems. Despite this potential, the low levels of residual monomer in clinical and commercial PVC use have not been shown to cause cancer (Vedanarayanan and Fernandez, 1987).

### Degradation of Vinyl Polymers

There are two main processes involved in the degradation of PVC: ‘zip’ dehydrochlorination and oxidation.

#### Zip Dehydrochlorination of PVC

‘Zip’ dehydrochlorination, depicted in Figure 5c, leads to the progression of double bonds along the length of the polymer chain (Shi et al., 2008; Singh and Sharma, 2008; Rabek, 2012) and occurs where there is already at least one double bond present in the length of the chain (Singh and Sharma, 2008; Rabek, 2012). As such, PVC with a high degree of polymerization which has more defects, is more susceptible to ‘zip’ dehydrochlorination (Shi et al., 2008). Polymeric C=C bonds readily absorb energy, which can transfer to a neighboring allylic (C-Cl or C-H) bond, causing the release of a chlorine free radical or hydrogen free radical. During the release of a chlorine free radical, the neighboring hydrogen atom may be released to from a double bond (-CH = CH-) and HCl molecule, or the chlorine free radical may escape cage recombination. In the case of the release of a hydrogen free radical, a β-chloro-alkyl radical (Figure 5b) is formed. This radical has a short lifespan as it readily releases a β-chlorine free radical to form a double bond (–CH = CH–).

This chlorine free radical, or those which have escaped cage recombination, is able to attack other allylic bonds, forming α-chloro-alkyl or β-chloro-alkyl free radicals (Figure 5b) and subsequent double bonds (Rabek, 2012). In the context of prosthetics, the formation of double bonds progressively degrades the color of PVC to a yellow and then dark red-brown, creating an obvious mismatch with native tissues (Shi et al., 2008; Rabek, 2012).

#### Oxidation of PVC

Oxidation of PVC occurs with the removal of hydrogen from PVC by a free radical, resulting in α-chloro-alkyl and β-chloro-alkyl free radicals (Figure 5b). These polymer alkyl radicals react with molecular oxygen, resulting in polymer peroxy radicals which subsequently remove hydrogen from neighboring allylic bonds or allylic bonds of other molecules (Rabek, 2012). As for zip’ dehydrochlorination, PVC oxidation also causes the PVC’s color to degrade to yellow before turning a dark red-brown. Additionally, the peroxy radicals become hydroperoxides which decompose to form ketones, aldehydes, acids, etc., which can lead to skin irritation of the person wearing the prosthesis (Shi et al., 2008; Rabek, 2012).

### Reinforcement of Vinyl Polymers

Polyvinyl chloride can be reinforced with a copolymer, polyvinyl acetate, to produce polyvinyl chloride acetate (PVCA). This copolymer is usually composed of 5–20% vinyl acetate polymers and copolymers. The advantages of reinforcing in this manner are improved stability to light and heat as well as lower temperature softening point. Other advantages over non-reinforced PVC are improved flexibility, chemical resistance, and heat and UV stability (Chalian and Phillips, 1974).

## Polyurethane Elastomer

In 1937, Otto Bayer discovered that diisocyanates and aliphatic diols (glycols) reacted to produce a material useful as a plastic or as a fiber (Covolan et al., 2004; Sharmin and Zafar, 2012). This material, called polyurethane (PU), is named such due to the urethane bond joining their monomers. As a prosthetic material, polyurethanes are useful as a bulk elastic polymer, as a liner, and as a foam.

### Chemistry of Polyurethane and Fabrication in Prosthetics

Since Bayer’s discovery, the synthesis of polyurethanes has expanded to include the reactions of many more isocyanates and diols (two hydroxyl groups)/polyols (multiple hydroxyl groups) to produce a large range of different physical properties through the combination of hard and soft segments (Chalian and Phillips, 1974; Goldberg et al., 1978; Craig et al., 1980; Affrossman et al., 1991; Covolan et al., 2004).

In the synthesis of polyurethanes, polyols can include aliphatic diols, hydroxyl terminated polyethers or polyesters. Longer chain polyethers and polyesters form the soft segments of the polymer chain. These are important in prosthetics to produce a soft skin-like feeling. Polyether polyols are preferable in the fabrication of soft tissue prosthetics; as they add flexibility, elasticity, softness, hydrophobicity, and resistance to hydrolytic degradation (Touchet and Cosgriff-Hernandez, 2016) while polyesters are susceptible to hydrolytic degradation with strong mechanical properties (Sharmin and Zafar, 2012; Touchet and Cosgriff-Hernandez, 2016).

The isocyanate groups, which compose the hard segments of the polymer chain, can be di or poly functional (Craig et al., 1980). They can also be aliphatic or an aromatic, with the aliphatic groups being more resistant against degradation due to UV exposure and hydrolysis, important for improved prosthetic service life (Chalian and Phillips, 1974; Craig et al., 1980). Aromatic groups, however, have stronger mechanical properties which may be given preference over UV stability (Touchet and Cosgriff-Hernandez, 2016).

Chain propagation (polymerization) occurs through the reaction between the polyol and isocyanate groups to result in a chain of hard and soft segments (Chalian and Phillips, 1974; Goldberg et al., 1978; Craig et al., 1980). Crosslinking between chains then occurs by trifunctional chain extenders, allophanate linkage, biuret linkage, and physical crosslinks on paracrystalline domains (Goldberg et al., 1978). It is possible to achieve this process of crosslinking at 100°C, allowing the use of dental stone molds and thereby lowering the cost of prosthetic production (Craig et al., 1980).

Often, diisocyanate-terminated pre-polymers are prepared in an initial stage, as shown in Figure 5d (Covolan et al., 2004). These pre-polymers are then joined through the use of a highly reactive diol chain extender/crosslinker (Goldberg et al., 1978; Covolan et al., 2004). This joins two isocyanate groups with a short diol producing a hard segment (Goldberg et al., 1978). The process allows for improved control over the chain sequence with longer soft segments produced as prepolymers before being connected by hard segments. The ability to tailor the segment lengths allows high degrees of control over the softness of the final prosthesis.

Polyurethane synthesis is stoichiometric, where the mass of the products equals the mass of the reactants, and therefore is a very sensitive technique (Goldberg et al., 1978; Craig et al., 1980; Aggarwal et al., 2016). Furthermore, the presence of moisture during this reaction will cause voids due to the release of carbon dioxide. These voids produce many unwanted effects such as altering the overall appearance and feeling of the prosthesis, reducing the reactivity of isocyanate groups which leads to incomplete polymerization, and producing polyureas which can irritate the skin of the wearer of the prosthesis (Chalian and Phillips, 1974; Goldberg et al., 1978; Craig et al., 1980; Affrossman et al., 1991). Organotin catalysts are another chemical used during synthesis to increase the rate of chain propagation (Goldberg et al., 1978; Craig et al., 1980). However, their effect can be neutralized by moisture which causes oxidation of catalysts. This, in turn, lowers the crosslink density and molecular weight of the final prosthesis (Goldberg et al., 1978).

When fabricating polyurethane foam, used in prosthetics where cushioning is needed (Rothman, 1962), the presence of moisture is not an error, but a necessity. The addition of both water and an emulsifier to the polyol-isocyanate reaction allows polymerization and the formation of gas to occur simultaneously. This results in desired voids within a gel structure (Rothman, 1962). The stiffness of the foam depends in part on the molecular structure of the chosen polyols. It is of note, that the stiffness of foam is not a constant as it undergoes multi-phase load-deformation (Todd et al., 1998).

Thermoplastic polyurethane elastomers can also be fabricated based on polycarbonates or polysiloxanes (Špírková et al., 2011; Liu et al., 2019). These additions can significantly change the mechanical and thermal properties of the polyurethane, in many cases improving their tensile strength and modulus and lowering the elasticity of the material. Polycarbonate based polyurethanes also generally possess improved resistance to organic solvents and are less sensitive to biodegradation (Eceiza et al., 2008). For example, the addition of polycarbonate nanoparticles into the polyurethane shows a distinctly segmented structure with strong changes seen in the tensile properties and large effects on dynamic mechanical thermal properties (Špírková et al., 2011). It is also possible to tailor the mechanical properties of the material by varying the molecular weights of the hard and soft segments. For example, synthesizing for a greater content of hard segments has been shown to increase the tensile modulus and decrease elongation at break (Eceiza et al., 2008). The properties of polycarbonate based polyurethanes lend themselves to orthopedic implant and cardiovascular prosthetics applications, however, they have found limited use in soft-tissue external prosthetics (Gostev et al., 2018). Polysiloxane based polyurethanes also have important biomedical engineering applications such as prosthetic dentistry, tissue engineering, breast prostheses and prosthetic linings, with good biocompatibility and material flexibility (González Calderón et al., 2019). Polyurethanes can have important application in prosthetics as a liner to interface between silicone prostheses and the patient’s skin. Silicones, as previously described, have many disadvantages including poor tear resistance at thin edges, vulnerability to microbial colonization, and absorption of facial oils (Grant et al., 2001; Deng et al., 2004; Chang et al., 2009). In 1987, Udagama (Udagama, 1987) studied the use of an aromatic polyether polyurethane film (Factor II, Inc.)^1^ to line silicone facial prostheses by preparing the silicone with Medical Adhesive Type A (Dow Corning Company) and the polyurethane sheet with S-2260 primer (Dow Corning Company) (Udagama, 1987; Deng et al., 2004; Kiat-amnuay et al., 2008; Chang et al., 2009; Patil et al., 2010; Shrivastava et al., 2015). The addition of the polyurethane liner improves the tear resistance of the thin regions of the silicone prosthesis, seals the silicone from absorbing oil, allows the use of water-based skin adhesives, increases surface smoothness thereby increasing comfort and ease of cleaning, and limiting microbial growth (Grant et al., 2001; Deng et al., 2004; Kiat-amnuay et al., 2008; Chang et al., 2009; Abd El-Fattah et al., 2013; Aggarwal et al., 2016). However, the methods of lining a silicone prosthesis with polyurethane methods have been described as lengthy and sophisticated (Abd El-Fattah et al., 2013) and Medical Adhesive Type A (Dow Corning Company) is known to produce acetic acid (an irritant) as it cures (Chang et al., 2009). In 1992, 8.0% of 88 respondents to a survey of American prosthetists (Andres et al., 1992) used this technique to line prostheses. A 2010 survey of 43 respondents found that 20.9% of respondents still used polyurethane lining for silicone prostheses with 41.9% of respondents having used it in the past (Montgomery and Kiat-Amnuay, 2010). The respondents of the 2010 survey used 0.05 mm polyurethane sheeting from Factor II, Inc. with PR-1205 (Dow Corning Company), Sofreliner (Tokuyama Dental America Inc., Encinitas, CA, United States), and A-330-G (Factor II Inc.) primers.

### Properties of Polyurethane

Polyurethane used in prosthetics is pigmentable, relatively environmentally stable, does not require plasticizers to achieve a low modulus of elasticity, has a high tensile strength, and has high tear resistance. However the reactions to produce polyurethane are stoichiometric, therefore difficult to work with, furthermore the material shows a yellow discoloration after aging (Chalian and Phillips, 1974; Goldberg et al., 1978; Craig et al., 1980). As discovered in a study by Leonhard et al. (2013), polyurethanes are also more susceptible to biofilm formation than silicone, potentially due to crack formation that occurs if the polyurethane becomes saturated with water.

The use of polyurethane as a liner for silicone prostheses is made more challenging due to difficulties adhering polyurethane with silicone layers. Although adherence can be improved through the use of a primer, it is still limited and prone to failure (Grant et al., 2001; Deng et al., 2004; Chang et al., 2009). This delamination has been shown to be repairable by Wu and Gerngross (2009), who reapplied polyurethane liner onto a silicone prosthesis.

### Degradation of Polyurethane

The use of polyurethane as a liner in silicone prostheses has been shown to improve the preservation of tear resistance, elasticity and tensile strength of silicone prostheses (Abd El-Fattah et al., 2013). The advantages of this polymer as a liner, however, are mitigated due to a heightened vulnerability of unwanted biofilm formation on polyurethane over silicone as shown in a study by Leonhard et al. (2013). The reason for this has been investigated by Boubakri et al. (2010) who found that, when immersed in water, micro-cracks appear in the surface. This is because polyurethane first follows a Fickian domain, such that the rate of absorption is proportional to the square root of time of immersion. During this time there is an initial plasticization effect; the water molecules increase flexibility and tensile properties of polyurethane as they diffuse between the molecular chains (Boubakri et al., 2010). However, as the level of absorption exceeds saturation, micro-cracks appear (Boubakri et al., 2010), forming ideal environments for the formation of biofilms.

## Chlorinated Polyethylene

In 1973, at a conference on the state of maxillofacial prosthetic materials held by the National Institute of Dental Research, the Gulf South Research Institute proposed that research be conducted into a variety of industrial rubber materials as potential maxillofacial prosthetic materials (Lemon et al., 2005). The institute received a grant to fund their research from 1976 to 1979 (May and Guerra, 1978; Lemon et al., 2005). During this time, a new prosthetic material made from thermoplastic chlorinated polyethylene (CPE) was formulated. This material appeared to have similar material properties to silicones, but was low cost and possessed thermoplastic properties such as the ability to be easily repaired, relined, reconditioned and reprocessed (May and Guerra, 1978; Kiat-amnuay et al., 2010). CPE is also more easily bonded than silicone and possesses a greater tear strength and surface wettability (May and Guerra, 1978; Kiat-amnuay et al., 2008). Further funding was obtained from 1983 to 1987 which enabled the formula to be refined and a small clinical trial at the Charity Hospital of New Orleans commenced (Lemon et al., 2005). In 2010, Kiat-amnuay et al. (2010) published a prospective, randomized, controlled, double-blind, single-crossover, multicentre, phase III clinical trial comparing maxillofacial prostheses made of CPE and medical-grade silicone. Amongst other findings, it was shown that while patients who were familiar with silicone prostheses found silicone to be superior in comfort and appearance, patients who were unfamiliar with silicone prostheses showed no preference between the two materials.

### Chemistry of Chlorinated Polyethylene and Fabrication in Prosthetics

Chlorinated polyethylene is produced by the controlled chlorination of high-density polyethylene in an aqueous slurry, such that the chlorination of the polymer chain occurs randomly. CPEs vary in chlorine content (approximately 25 to 42%), molecular weight and crystallinity (Manaila et al., 2012).

In order to use CPE as a material in prosthetic fabrication, it must be processed on heated mills into large sheets. During this process, intrinsic colorants may be added as needed to match the pigment color of the patient (Kiat-amnuay et al., 2008, 2010). To further improve the aesthetics of the final prosthesis, the sheets can also be processed with red rayon flocking to appear as capillaries on the surface (Kiat-amnuay et al., 2010). The CPE and mold are then heated to 110–115° (Kiat-amnuay et al., 2008, 2010; Eleni et al., 2009c, 2011c) or placed in a pressure cooker at approximately 60kPa (equivalent to 115°C) (Kiat-amnuay et al., 2008, 2010) for 10 min. This necessitates that the mold must be placed into a metal flask to prevent fracture (Kiat-amnuay et al., 2008, 2010). After this, more CPE is added and the process repeated until the mold is sufficiently filled (Kiat-amnuay et al., 2010). Kiat-amnuay et al. (2010), in their clinical trial, found that prosthetic technicians criticized CPE prostheses as more complex, harder to manipulate, having more flaws, and more likely to break during fabrication when compared with conventional silicone prostheses. These criticisms were formed even though the overall time to fabricate CPE prostheses was reported to be shorter than that for silicone (Kiat-amnuay et al., 2010).

### Properties of Chlorinated Polyethylene

Chlorinated polyethylene is a thermoplastic with applications as a maxillofacial prosthetic material as an alternative to silicones, partly due to its low cost and thermoplastic properties (May and Guerra, 1978; Kiat-amnuay et al., 2010). As a thermoplastic, CPE can be repaired, relined, reconditioned, and reprocessed in a short time for small corrections. CPE can also be used with a wider variety of adhesives than silicone, and has much greater tear strength, and surface wettability comparable to skin (May and Guerra, 1978; Kiat-amnuay et al., 2008). In addition, CPE is very low in toxicity, non-carcinogenic, less irritating to the mucosa than silicone, and does not support fungus growth (Kiat-amnuay et al., 2010). The drawbacks of CPE in soft tissue prosthetics, however, is that prostheses of CPE tend to have thicker borders and are more difficultly matched to the skin color and texture of the patient (Kiat-amnuay et al., 2010).

### Degradation of Chlorinated Polyethylene

Like other polymers used in prosthetics, CPE is also susceptible to degradation over time. Outdoor weathering has been found to affect CPE by increasing maximum stress and strain while decreasing the elastic modulus (compression and tensile), yield stress and strain, hardness, and glass transition temperature (Eleni et al., 2009b, d, 2011c). Unlike silicone, which undergoes crosslinking over time (becoming harder), nuclear magnetic resonance and infrared spectroscopy studies have found that CPE mainly undergoes chain scission reactions during photo-oxidative degradation, leading to softening of the polymer (Eleni et al., 2009b, d, 2011c).

The relative newness of this polymer means there are few studies on the effect of skin secretions on its aesthetic and mechanical properties. While simulated perspiration has been found to increase the elastic modulus, hardness, and the weight of CPE to make it less skin like; simulated sebum decreases the elastic modulus, hardness, and weight (Eleni et al., 2009c). It is theorized that while simulated perspiration (an aqueous solution) causes water absorption and the propagation of crosslinking reactions, simulated sebum (a fatty solution) interacts with the surface of the polymer to extract compounds (Eleni et al., 2009c). Maximum stress and strain, however, are not significantly affected by either solution. Color, on the other hand, is significantly changed by both simulated perspiration and sebum, more greatly by simulated sebum. This is not ideal for a prosthesis that must blend in with native tissues. Glass transition temperature was increased by simulated perspiration; and melting temperature was increased in both solutions (Eleni et al., 2009c).

The effect of different disinfection methods is more unclear. Eleni et al. published two papers in 2013 (Eleni et al., 2013a, b), investigating the effects of microwave disinfection, sodium hypochlorite solution, neutral soap, and commercial disinfectant on CPE. The two papers found contrasting results on whether sodium hypochlorite solution caused an increase (Eleni et al., 2013b) or a decrease (Eleni et al., 2013a) in hardness, suggesting that more work is needed to understand the material.

## Conclusion

Polymers in soft tissue prosthetics are life-changing for most people affected by disfigurement by restoring function and aesthetics. The key challenge is to replicate all unique properties of natural living tissue using these synthetic polymer materials. Furthermore, with the prosthetic polymers’ exposure to UV light, salt water, make up and skin secretions, it is vital to understand and control physical, chemical, biological and aesthetic changes in the polymers over time to ensure patients are provided with the best possible improvement in their quality of life.

From simple woods and metals used as prosthetics thousands of years ago to composite polymers, the progression of materials science has seen impressive advancements. Although there is no perfect material available for all applications, consideration must be given to the aesthetics, attachment, fabrication, robustness and the wellbeing of the patient. Prostheses for various regions of the body also require unique considerations, mimicking as closely as possible their unique anatomies and environments.

Commonly used prosthetic materials possess an impressive array of characteristics. Today represents the crossroad in materials development and fabrication techniques as new 3D healthcare technologies begin to replace traditional hand-crafting techniques. This will revolutionize the aesthetics and function of prostheses themselves, and lead to new innovations that provide even greater realism and lower costs. These 3D manufacturing technologies and new techniques will drive down healthcare costs to bring the goal of universal access to better polymer prostheses closer to the patient. Though developments in tissue-engineered solutions are posed to replace the use of these temporary external prostheses, there will always be a role for external prostheses, either as a temporary or more affordable solution to restoring facial aesthetic. These tissue-engineered implants will employ a different range of biopolymers; such as polycaprolactone, polylactic acid, and polyglycolic acid; to meet a different set of requirements in the fabrication of 3D printed tissues (Ding et al., 2019; Zhang et al., 2019). The future of prosthetics, developed through the close collaboration between researchers, industry, healthcare workers and patients, will continue to provide better solutions and ensure improved quality of life for millions of people around the world.

## Author Contributions

MW and SP contributed to the conception of the manuscript. RC and MR researched and wrote the first draft and revised the manuscript. MW and SP structured, reviewed, and revised the manuscript. All authors contributed to manuscript revision, and have read and approved the submitted version.

## Conflict of Interest

The authors declare that the research was conducted in the absence of any commercial or financial relationships that could be construed as a potential conflict of interest.

## Footnotes

1 https://solutions.covestro.com/en/brands/dureflex
